# Correction to “A ‘Glympse’ Into Neurodegeneration: Diffusion MRI and Cerebrospinal Fluid Aquaporin‐4 for the Assessment of Glymphatic System in Alzheimer's Disease and Other Dementias”

**DOI:** 10.1002/hbm.70036

**Published:** 2024-10-01

Sacchi, L., F. D'Agata, C. Campisi, et al. 2024. “A ‘Glympse’ Into Neurodegeneration: Diffusion MRI and Cerebrospinal Fluid Aquaporin‐4 for the Assessment of Glymphatic System in Alzheimer's Disease and Other Dementias.” *Human Brain Mapping* 45, no. 12: e26805. https://doi.org/10.1002/hbm.26805.

The following funding information was missing from the Acknowledgments section: Open access publishing facilitated by Universita degli Studi di Milano, as part of the Wiley—CRUI‐CARE agreement.

The online version of this article has been corrected accordingly.

